# Wolf Media Coverage in the Region of Castilla y León (Spain): Variations over Time and in Two Contrasting Socio-Ecological Settings

**DOI:** 10.3390/ani10040736

**Published:** 2020-04-23

**Authors:** Miguel Delibes-Mateos

**Affiliations:** Instituto de Estudios Sociales Avanzados (IESA-CSIC), Campo Santo de los Mártires 7, 14004 Córdoba, Spain; mdelibes@iesa.csic.es; Tel.: +34-957760527

**Keywords:** *Canis lupus*, content analysis, human-wildlife conflicts, livestock loss, wolf attacks

## Abstract

**Simple Summary:**

Wolf management is often controversial, because this carnivore is viewed by some as a major threat for livestock, while others perceive it as a flagship for environmental conservation. Attitudes towards the wolf can be influenced by how the media portrays this canid, and media analysis can therefore be a useful tool for understanding and managing conflicts over wolf management. My aim was to study wolf media coverage in a newspaper in northern Spain, how it varied over the period 2006–2017, and in two different socio-ecological settings. Most documents focused on the conflictive relationship between the wolf and livestock, particularly in the south of the study area, where the carnivore is protected and has recolonised new localities, causing increasing damage to livestock. In the north, where wolves have been present for a long time and are a game species, wolf media coverage was more diverse and addressed other topics such as wolf conservation or hunting more frequently. In conclusion, this study suggests that the media often portrays the wolf as a risk for livestock and thus for human livelihood in northern Spain, which could have a significant influence on public attitudes towards the species and potentially compromise coexistence between wolves and humans.

**Abstract:**

People’s attitudes towards large carnivores, and thus public support for their conservation, can be influenced by how these species are framed in the media. Therefore, assessing media coverage of large carnivores is of particular interest for their coexistence with humans. I used content analysis to assess how the grey wolf was portrayed in a newspaper in northern Spain, how wolf media coverage varied over time (2006–2017), and in two different socio-ecological settings. Most documents addressed the conflictive relationship between the wolf and livestock (60%; n = 902). Moreover, coverage of this relationship increased over the study period in the south of the study area, where the wolf is strictly protected, has recolonised new localities, and damage to livestock has increased. Overall, other topics, such as wolf conservation or hunting, appeared much less frequently in the media, but predominated in the north of the study area, where the wolf is more abundant and huntable. Conflictive issues like wolf-livestock interactions are generally attractive for audiences, but drawing attention to this issue may compromise the management of conflicts associated with wolves. Ideally, the media should promote potential wolf conservation values if coexistence between wolves and humans is sought.

## 1. Introduction

Large carnivores have substantially recovered over the past decades in humanised landscapes of North America and Europe [1]. In these areas, conflicts over large carnivore management have become increasingly common: some people support large carnivore conservation, while others advocate their killing, mainly because of livestock loss caused by depredation [2]. In this context, understanding the relationships between humans and large carnivores is important to ensure their coexistence [3]. 

People’s attitudes towards large carnivores may influence their support for specific management policies and practices aimed at large carnivore restoration [4,5]. Such attitudes are often influenced by the extent to which people are informed about carnivores, as information can affect people’s personal beliefs, opinions, values, or emotional states [6]. Most people are likely to receive information on wildlife from the media [7,8,9]. Therefore, assessing media coverage of large carnivores is of great interest for their coexistence with humans. 

The media plays an agenda-setting role by highlighting certain issues in the coverage of possible incidents and developments [10]. This can lead the audience to develop a media-induced view of large carnivores. In this sense, evaluating the topics relayed in the media and how they are portrayed is critical for gaining insight into interactions between humans and large carnivores [2,11]. It is also important to track these topics over time, because it may help identify factors that influence the frequency of exposure of each topic in the media [12]. In this regard, content analysis is a very useful method for identifying the main topics relayed in newspaper articles and is increasingly used in studies that address the media coverage of large carnivores [2,9,13]. 

The grey wolf (*Canis lupus*) often attracts considerable media attention [14] because debates about its presence, impacts, and management are very often contentious [15,16]. This is likely the reason why wolf media coverage has been increasingly investigated (e.g., [2,17]). Recent research has demonstrated that the importance the media place on different wolf-related topics changes both in space and over time. In France, for example, the topics employed to portray wolves were found to differ in regional and national newspapers [2]. In addition, wolf-related topics varied in North America following changes in wolf conservation status [12]. As these previous studies suggest, wolf media coverage is dynamic and context-specific. 

My main goal in this study was to explore the evolution of topics surrounding the wolf in a newspaper published in Castilla y León (northern Spain), the region with the largest number of wolf packs in the country [16]. My goals also included assessing variations in wolf framing over time, between different areas and across articles featuring wolves as a primary or secondary topic. In Castilla y León, there exist two very contrasting situations in terms of wolf management, policy, and ecology. The wolf is strictly protected in the part of the region below the River Douro, while it is a game species in the north. Although the number of wolves is considerably higher in the north [18], most reported attacks on livestock take place in the south [19] where wolves have colonised some new areas in recent years [16]. I suspected that these notable differences may have resulted in variations in wolf media coverage between sub-regions. In addition, I expected that more controversial topics (e.g., wolf-livestock interactions) would occur more frequently in documents in which the wolf was the central theme, while less controversial issues would also receive attention in documents in which the wolf was not the central theme. 

## 2. Materials and Methods

### 2.1. Study Area and Context

**Figure 1:**
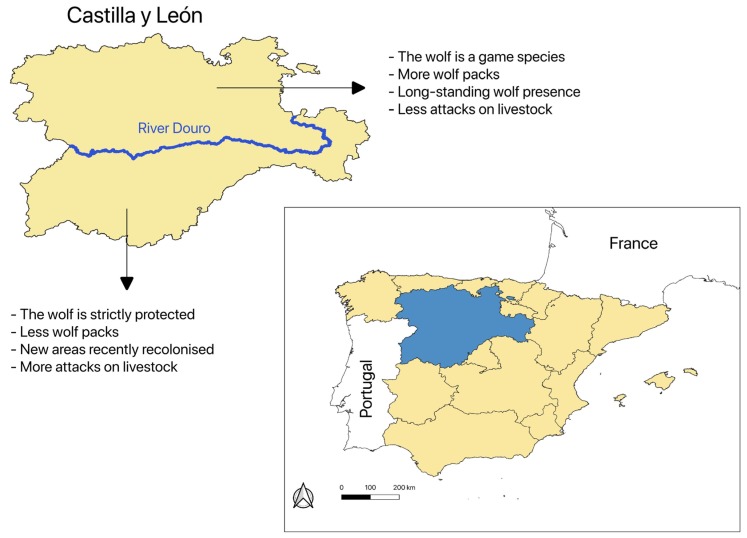
Location of the Castilla y León region in northern Spain. The River Douro divides the region in two sub-regions with highly contrasting situations in terms of wolf management, policy, and ecology.

The region of Castilla y León is located in the north-western Iberian Peninsula (Figure 1). Spanning 94,226 km^2^, it is the largest region of Spain. The human population density of Castilla y León is low (~25 inhabitants/km^2^). Plains in the centre of the region are mostly dedicated to agriculture and are surrounded by mountain ranges dominated by woodlands [20]. The mountain ranges act as a barrier to maritime influences, lending most of the region a continental climate with long cold winters and warm summers. In terms of precipitation, the region is mainly characterised by equinoctial rains and summer droughts [21]. 

The EU Habitats Directive confers strictly protected Annex IV status and more flexible Annex V status on wolves occurring south and north of the River Douro, respectively [22]. According to this legal framework, wolf hunting is authorised in the north, but not in the south (Figure 1), where a few individuals are culled every year by rangers of the official environmental agency to reduce social tensions. Both farming and hunting associations, as well as the regional government, have repeatedly requested the EU and the Spanish government to declare the wolf as a game species also in the southern part of Castilla y León. Castilla y León holds >50% of the Spanish wolf packs [16]. In 2012–2013, there were 152 and 27 packs in the north and the south of the River Douro, respectively [18]. In the study area, wolf attacks on livestock are relatively frequent. For example, more than 5100 attacks were reported between 2005 and 2012 [23]. Wolf attacks substantially increased to the south of the River Douro from 2007 to 2017, while the increase was only moderate in the north. In 2017, for example, >73% of all attacks (n = 1989) occurred in the south [19]. The regional government compensates farmers financially for the damage wolves cause to livestock. In addition, the government provides farmers funding for fences and guard dogs upon request.

### 2.2. Content Analysis

In March 2018, I searched all the documents containing the word “lobo” (wolf in Spanish) published in the digital archives of daily issues of the main regional newspaper, *El Norte de Castilla*. Digital archives of the periodical dating from 2006 are available. In 2019, the newspaper had an estimated readership of 144,000 daily readers (http://reporting.aimc.es/index.html#/main/diarios), ranking first of all general-interest newspapers in Castilla y León. *El Norte de Castilla* publishes different editions in four provinces (Salamanca and Segovia to the south of the River Douro and Palencia and Valladolid to the north), although it covers the entire region. I reviewed all the documents and removed those in which the word “wolf” did not refer to the animal [2,12]. For the analyses, I differentiated between: (1) documents that referred to the north and south of the River Douro and those that could not be assigned to any of the aforementioned categories, that is, those that addressed wolf-related topics at larger spatial scales (e.g., national or European) or those that did not specify any spatial area (i.e., generic documents); (2) documents in which the wolf was the central theme of the article (primary articles) and those in which the canid was not the main focus (secondary articles). The latter included, for example, articles that described the regional hunting quotas for all game species, including the wolf, or documents that highlighted the natural value of a particular area, where the presence of wolves was only one of the attributes of the area’s natural value.

Data analysis was inductive as data codification was carried out by establishing categories from the data itself [24]. In other words, I identified seven main topics related to the wolf during the initial exploration of the data, and then used the data for the codification of the text material in Nvivo 10 software (QSR International Pty Ltd., 2012, Melbourne, Austrilia). The topics were as follows. (1) Wolf-livestock interactions: the documents coded in this topic referred either to wolf attacks on livestock, management options to reduce or compensate livestock losses caused by wolves (e.g., fencing, guardian dogs, culling by official rangers, compensation schemes, etc.), farmers’ claims for wolf attacks on livestock or policymakers declarations of intention to seek solutions for livestock damage caused by wolves. (2) Wolf conservation: this topic was used for documents that highlighted the need to conserve the wolf or those that made reference to factors that may threaten the conservation of this top predator. Among others, these documents included references to conservationists’ claims about the negative effects of hunting on wolf conservation or the potential risks that the construction of human infrastructures like roads may have for wolves. (3) Wolf-livestock coexistence: this topic was used for documents that made reference to both wolf conservation and wolf-livestock interaction. For example, some documents discussed the regional wolf management plan, whose main goal is to promote the coexistence of wolf occurrence and conservation with traditional rural activities such as livestock raising [25]. (4) Hunting: the documents coded in this topic referred to the wolf as a game species. These included, for example, documents about the number of wolves that the government allowed hunters to kill in a particular hunting season or the monetary income generated by wolf hunting in some areas. (5) Poaching: I assigned this topic to documents that showed cases of illegal killing of wolves, including poisoning, illegal shooting, trapping, etc. (6) Natural value: this topic was used for documents that portrayed the wolf as an attraction for tourists and documents that described the natural value of some areas and referred specifically to wolf occurrence as one of the main assets of such areas. (7) Dissemination: the documents coded in this topic referred to activities that aimed to disseminate knowledge about the wolf, including conferences, courses, or exhibitions. Documents that did not address any of these topics were coded as “Others”. These included, for example, articles about wolf ecology and evolution or the cultural meaning of wolves. Although each document generally only addressed one of these topics, in a few cases two or more topics were assigned to a particular document. 

### 2.3. Statistical Analysis

Chi-square tests were performed for the statistical analysis. Specifically, the tests were used to determine: (1) variations in the frequency of documents by area covered (the variable AREA had three levels: north of the River Douro, south of the River Douro, and non-assignable; (2) differences between the frequency of primary and secondary articles (the variable PROTA had two levels: the wolf as the central theme and the wolf as secondary in importance in the document; (3) variations in the frequency of documents published per year (YEAR was divided into 12 levels from 2006 to 2017). In addition, differences in frequencies of articles published per year between each area and between the wolf being the central theme of the documents or not were investigated using Chi-square tests for contingency tables (AREA: 12 × 3; PROTA: 12 × 2; see an example in [26]).

Given the binary nature of the variables (1 = the topic was addressed in a particular document; 0 = the topic was not addressed), logistic regressions were used to test for associations between the topics dealt with in the documents (see Results) and AREA, PROTA, and YEAR. One model was fitted for each of the topics. Collinearity among categorical factors was assessed (i.e., AREA and PROTA) using Cramér’s V index of correlation (R function “assocstats”, package “vcd”). This correlation index is based on Chi-square tests for categorical variables that include >2 levels [27]. Collinearity was not an issue among the categorical variables (coefficient: YEAR/PROTA = 0.32), and therefore both predictors were included in the models (see an example in [6]). YEAR was included in the models as a continuous variable in order to test the effect of time on the binomial responses. 

## 3. Results

A total of 3906 documents contained the word “wolf”, but only 902 referred to the animal. 

### 3.1. Variations in Wolf Presence in the Media 

**Figure 2:**
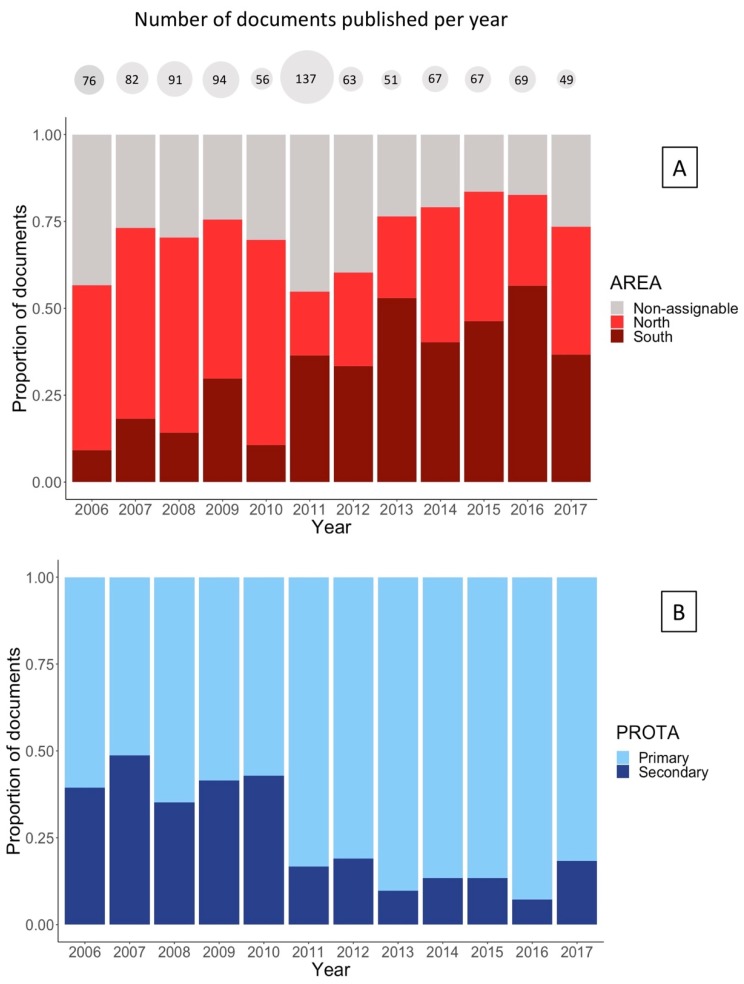
(**A**) Proportion of documents published annually that referred to the three categories of areas considered: north of the River Douro, south of the River Douro, and non-assignable (i.e., larger spatial scales or generic documents). (**B**) Proportion of documents published annually according to whether the wolf was the central theme of the article (primary) or not (secondary). The total number of documents published each year is shown in grey circles above the figure.

The number of documents per year on wolves was slightly higher in the period 2006–2009 and then decreased, with the exception of 2011, when a noticeable peak was detected (Figure 2). Overall, there were significantly more documents referring to the north of the River Douro (n = 349) than to the south (n = 282) and to non-assignable areas (n = 271) (Chi-squared test for given probabilities: χ^2^ = 11.856, df = 2, *p* = 0.002). The frequency of publication of wolf-related documents in the three areas (north, south, and non-assignable) varied significantly over the study period (Pearson’s chi-squared test: χ^2^ = 135.45, df = 22, *p* < 0.001). Documents referring to the north of the River Douro predominated in the first part of the study period (2006–2010), while they were less common than those referring to the south of the River Douro from 2011 onward, except in 2017 when the proportion of documents for both areas was practically the same (Figure 2A). 

In addition, there were more documents in which the wolf was the central theme of the article (primary and secondary articles: 665 and 237, respectively; Chi-squared test for given probabilities: χ^2^ = 203.09, df =1, *p* < 0.001). The frequency of publication of primary and secondary wolf articles varied significantly during the study period (Pearson’s chi-squared test: χ^2^ = 92.263, df = 11, *p* < 0.001). Primary articles increased across the study period, clearly predominating from 2011 onwards (Figure 2B).

### 3.2. Variations in Media Portrayal of the Wolf

In the period 2006–2017, most documents (n = 549) addressed the conflictive relationship between the wolf and livestock. Wolf conservation, hunting, and the natural value of the species were covered in 107, 103, and 98 documents, respectively. The rest of the topics were even less present in the media (58 documents on poaching, 51 others, 40 on the coexistence between wolf conservation and livestock production, and 39 related to dissemination).

**Figure 3:**
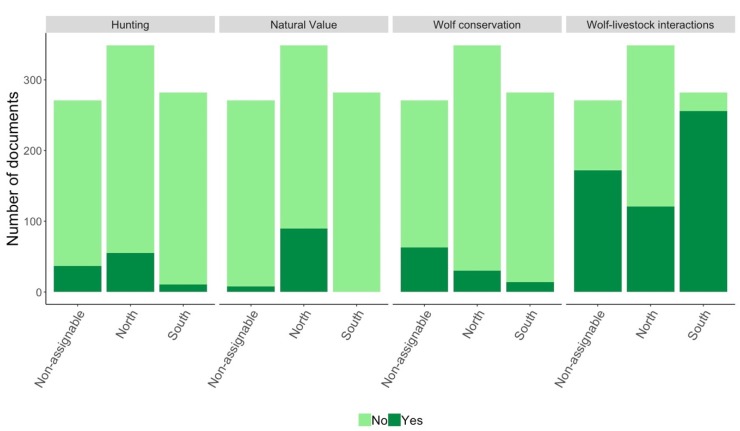
Number of documents published on the four main topics identified based on the three categories of areas considered: north of the River Douro, south of the River Douro, and non-assignable (i.e., larger spatial scales or generic documents).

**Figure 4:**
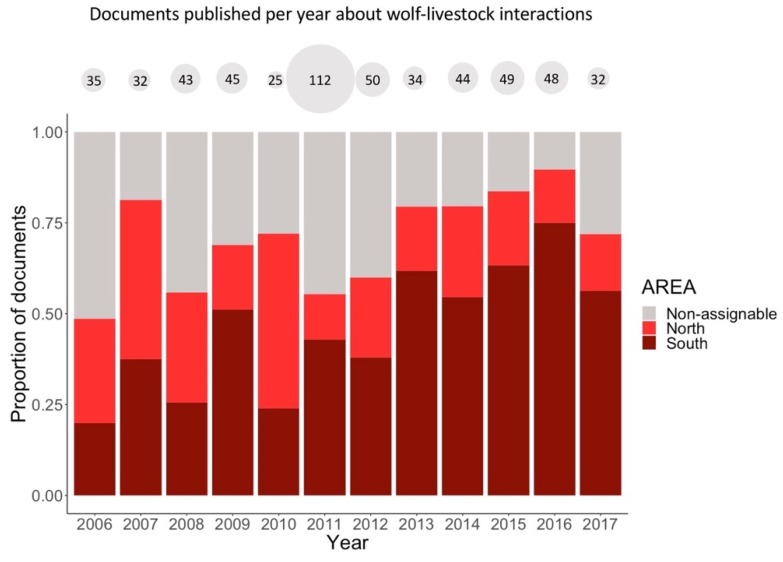
Proportion of documents published annually about wolf-livestock interactions according to the three categories of areas considered: north of the River Douro, south of the River Douro, and non-assignable (i.e., larger spatial scales or generic documents). The total number of documents published every year on wolf-livestock interactions is shown in grey circles above the figure.

The topic of wolf-livestock interaction was covered more widely in the area to the south of the River Douro and to a lesser extent in the non-assignable area than in the north of the River Douro (Table 1; Figure 3). Wolf conservation was more frequently addressed in generic documents and in those that referred to the region as a whole (i.e., non-assignable area). In addition, this topic was more frequent in the north than in the south of the River Douro, although the differences were not statistically significant (Table 1; Figure 3). In general, the topics of wolf hunting, poaching and the natural value of wolves appeared more often in documents referring to the north of the River Douro (Table 1; Figure 3). The topic wolf-livestock coexistence was covered more frequently in the non-assignable area (Table 1). The topics wolf-livestock interaction and wolf-livestock coexistence were mostly addressed in primary articles, while the topics of wolf hunting, poaching and dissemination were covered more often in secondary documents (Table 1). The topic hunting and other documents increased over the study period while natural value, dissemination and conservation decreased; although for the topic conservation, the variable YEAR was only marginally significant in the model (Table 1). Wolf-livestock interaction was more frequently addressed in documents that referred to the north of the River Douro and to non-assignable areas between 2006 and 2010. In contrast, this topic predominated in documents that made reference to the south of the River Douro in the second half of the study period (Pearson’s chi-squared test: χ^2^ = 81.22, df = 22, *p* < 0.001; Figure 4). 

## 4. Discussion

### 4.1. Conflictive Media Portrayal of the Wolf

News values provide editors and journalists a repertoire of story slants and news hooks they can employ in presenting a particular issue to readers [28]. Conflict, human interest, and consequence are included in any list of basic news values [29]. MacDougall argued that conflict is one of the most significant and reliable contributors to readers’ interest [28]. In this sense, wildlife topics generally appear most often in the media when wildlife causes problems for humans [30,31]. In agreement with this, most of the articles analysed in this study focused on conflictive interactions between wolves and livestock. This suggests that journalists may find conflict to be a useful storytelling angle for documents related to wolves in Spain as occurs in other regions (e.g., [17]). Second, describing the individuals affected by unfortunate events can arouse sympathetic interest [28]. From this perspective, the personal story of livestock breeders who have been impacted by wolves may be much more attractive for readers than other issues. Previous studies conducted in France and the US revealed that this human-centred viewpoint of wolf articles is very common in local and regional newspapers [2,12], such as the one examined here. In addition, local events are generally of greater consequence and interest to audiences [28]. Many of the articles analysed in this work described wolf attacks on livestock near villages and often reported the views of the affected farmers. This suggests that what occurs locally may be more attractive for readers of a regional newspaper like *El Norte de Castilla* than other more global issues (see also [2]).

### 4.2. Variations between Regions 

Novel exposures to carnivores during their recolonization can cause greater damage to unprotected livestock. As a result, these events often receive wider media attention and contribute to polarising the discussion [14,32]. This coincides with my findings, which show that nearly all of the articles focusing on the south of the Castilla y León region, where wolves have recently colonised new areas and caused increasing livestock losses [16,19], dealt with the relationships between the canid and livestock. The fact that the wolf was the main focus in most of these documents and that the wolf attracted disproportionate attention in relation to its abundance in the south of the River Douro may also point to the problematic media portrayal of the canid in this part of the study area. This closely resembles the findings in the US, where newspaper stories in states with new wolf populations had the greatest number of negative attitude expressions towards the wolf [17]. In my study, media coverage was more diverse in the north of the study area, where the species has a long-standing presence. The higher frequency of topics like wolf hunting and the natural value of wolves in this part of the region may reflect the fact that the wolf constitutes an attractive resource for hunters and wildlife watchers in the area. In the state of Idaho, articles on topics related to hunting tripled in salience over the 20 years after wolves were reintroduced [12]. I did not observe this pattern in the south of Castilla y León after wolf recovery, likely because wolf hunting is currently not allowed in this area and/or because wolf presence is relatively recent in this area. 

### 4.3. Variations over Time

Interestingly, wolf colonisation of new areas in northern Spain and the subsequent increase in attacks on livestock did not result in higher overall wolf media coverage. In fact, the total number of articles published per year slightly decreased in the second half of the study period. However, I found that the north of the River Douro received more media attention in the first part of the study period, while the focus changed to the south in the second half. Moreover, the annual publication of articles describing conflictive relationships between wolves and livestock increased over time in the area south of the River Douro (Figure 4). This growth was particularly noteworthy in 2011, when confirmed livestock depredations by wolves began to increase consistently in the area [19]. In 2011, a similar increase was also observed in documents referring to other areas (Figure 4). In that year, both national and regional elections were held in Spain. Following the elections, representatives of the Castilla y León regional government repeatedly pressured the recently constituted national government to request the EU to withdraw wolf protection in the south of the River Douro. The specific intention of this media pressure was to declare the wolf a game species and thus control the species for the benefit of livestock breeding. In fact, this continues to be a heated debate in the local and regional newspapers of Castilla y León. Additionally, the overall increase in articles portraying the wolf as problematic in this area might reflect the dramatization of these events, which could potentially prevent the development of a constructive dialogue and result in conflicts becoming increasingly intractable [33]. 

It is striking that the salience of topics related to wolf conservation and their natural value declined between 2006 and 2017. The former may be linked to wolf recovery not only in part of the study area, but also in some neighbouring Spanish regions [16]. The latter is more surprising as wolf colonisation of new areas could be viewed as an opportunity to attract environmental tourism [34]. Importantly, none of the articles portrayed the wolf as a potentially valuable natural resource in the south of the River Douro (Figure 3). 

### 4.4. Media Coverage and Human–wildlife Conflicts

Human–wildlife conflicts are increasing in frequency and magnitude [35], and the media is an important channel for conflict management due to its potential role as both an actor and facilitator of discussions [26]. Public outcry often has much more to do with perceptions of potential risks than actual crop, livestock, or property loss [36], and the media usually influences people’s perceptions of risks associated with large carnivores [30]. My study reveals that the media often portrays the wolf as a risk for livestock and thus for human livelihoods in northern Spain, which could have a significant influence on public attitudes towards the species. Media campaigns can influence people’s behaviour [37], and therefore could be used to encourage the stakeholders involved in the wolf-livestock conflict (i.e., farmers and environmentalists) to seek coexistence and advocate to policymakers the benefits of making livestock raising and wolf conservation compatible. In addition, my results suggest that the topics become increasingly diversified with length of coexistence with wolves, and therefore, from a conservation perspective, the objective should be to de-dramatize wolf media coverage after wolf recolonization. This could be achieved via the proactive engagement of scientists with journalists and could be anticipated in regions where wolves have not yet recolonised. 

### 4.5. Shortcomings and Way Forward

In media content analysis an external check is typically used to avoid potential subjectivity bias when only one evaluator categorises texts (e.g., [12]). As this was not done in the present study, some misinterpretation of the textual data might have occurred. Nevertheless, given that the topics identified in this study were very general (and thus easily identifiable) and well differentiated (and thus hardly mistakable), I would not expect that incorrect topic assignations during text codification were frequent. A limitation of this study is that it is based on only one regional newspaper. Future studies should assess if similar findings are found in local and national newspapers, as these could frame the wolf differently [2]. In addition, wolves occur in relatively high numbers in other regions of northern Spain like Galicia or Asturias, which have their own peculiarities regarding wolf management [16]. Media content analyses in those regions, as well as in Portugal, where the wolf is strictly protected, would be highly useful to investigate the diversity of wolf media coverage in the Iberian Peninsula. Finally, individuals and organisations currently make use of powerful communication tools provided by the Internet (e.g., blogs, websites, or social media platforms) to spread their influence directly to thousands of people [38]. It would be interesting for future investigations to also focus on assessing how the wolf is portrayed in such communication channels, and how different collectives interact on the Internet. 

## 5. Conclusions

My study shows that the media often portrays the wolf as a risk for livestock and thus for human livelihood in northern Spain. Conflictive issues like wolf-livestock interactions are generally attractive for audiences, but drawing attention to this issue could have a significant influence on public attitudes towards the species and potentially compromise coexistence between wolves and humans. Ideally, the media should promote potential wolf conservation values if coexistence between wolves and humans is sought.

## Figures and Tables

**Table 1:** Results of logistic regressions used to test for associations between the topics identified in the documents and AREA (north of the River Douro, south of the River Douro, and non-assignable documents), PROTA (primary and secondary articles), and YEAR (2006–2017). The number of documents on each topic is shown in brackets. *** *p* < 0.001; ** *p* < 0.01; * *p* < 0.05.

	**Wolf-livestock Interaction (549)**			**Wolf Conservation (107)**			**Hunting (103)**			**Natural Value (98)**		
	**Estimate (SE)**	***Z*-Value**	**Pr (>|z|)**	**Estimate (SE)**	***Z*-Value**	**Pr (>|z|)**	**Estimate (SE)**	***Z*-Value**	**Pr(>|z|)**	**Estimate (SE)**	***Z*-Value**	**Pr (>|z|)**
Intercept	−82.34 (51.25)	−1.6	0.1	128.15 (70.24)	1.82	0.06	−215.98 (67.86)	-3.18	**	202.14 (76.48)	2.64	**
Year	0.04 (0.02)	1.57	0.11	−0.06 (0.03)	−1.86	0.06	0.1 (0.03)	3.16	**	−0.1 (0.03)	−2.65	**
Area: N/A	1.29 (0.18)	7.16	***	1.18 (0.24)	4.92	***	−0.17 (0.23	−0.75	0.45	−2.43 (0.38)	−6.41	***
Area: south	2.56 (0.24)	10.6	***	−0.54 (0.34)	−1.57	0.11	−1.46 (0.35)	−4.12	***	−18.35 (635.92)	−0.02	0.97
Primary: Yes	1.4 (0.18)	7.45	***	0.29 (0.24)	1.18	0.23	−0.82 (0.23)	−3.52	***	0.12 (0.24)	0.51	0.6
	**Poaching (58)**			**Wolf-Livestock Coexistence (40)**			**Dissemination (39)**			**Others (51)**		
	**Estimate (SE)**	***Z*** **-Value**	**Pr (>|z|)**	**Estimate (SE)**	***Z*** **-Value**	**Pr (>|z|)**	**Estimate (SE)**	***Z*** **-Value**	**Pr (>|z|)**	**Estimate (SE)**	***Z*** **-Value**	**Pr (>|z|)**
Intercept	−17.97 (88.3)	−0.2	0.83	57.68 (108.28)	0.53	0.59	329.92 (124.52)	2.65	**	−283.37 (91.58)	−3.13	**
Year	0.008 (0.04)	0.18	0.85	−0.03 (0.05)	−0.57	0.56	−0.16 (0.06)	−2.66	**	0.14 (0.04)	3.11	**
Area: N/A	−0.28 (0.3)	−0.93	0.35	1.81 (0.43)	4.21	***	−0.72 (0.4)	−1.78	0.07	0.36 (0.3)	1.19	0.23
Area: south	−1.1 (0.44)	−2.47	*	−0.86 (0.7)	−1.23	0.21	−0.61 (0.49)	−1.23	0.21	−1.43 (0.51)	−2.78	**
Primary: Yes	−0.8 (0.29)	−2.72	**	1.38 (0.49)	2.78	**	−0.75 (0.35)	−2.11	*	−0.45 (0.32)	−1.42	0.15

## References

[1] Chapron G., Kaczensky P., Linnell J.D.C., von Arx M., Huber D., Andrén H., López-Bao J.V., Adamec M., Álvares F., Anders O. (2014). Recovery of large carnivores in Europe’s modern human-dominated landscapes. Science.

[2] Chandelier M., Steuckardt A., Mathevert R., Diwersy S., Gimenez O. (2018). Content analysis of newspaper coverage of wolf recolonization in France using structural topic modelling. Biol. Conserv..

[3] Woodroffe R., Thirgood S., Rabinowitz A. (2005). People and Wildlife, Conflict or Coexistence?.

[4] Bruskotter J.T., Vaske J.J., Schmidt R.H. (2009). Social and cognitive correlates of Utah residents’ acceptance of the lethal control of wolves. Hum. Dimens. Wildl..

[5] Wilson R.S., Bruskotter J.T. (2009). Assessing the impact of decision frame and existing attitudes on support for wolf restoration in the United States. Hum. Dimens. Wildl..

[6] Arbieu U., Mehring M., Bunnefeld N., Kaczensky P., Reinhardt I., Ansorge H., Böhning-Gaese K., Glikman J.A., Kluth G., Nowak C. (2019). Attitudes towards returning wolves (*Canis lupus*) in Germany: Exposure, information sources and trust matter. Biol. Conserv..

[7] Barua M. (2010). Whose issue? Representations of human-elephant conflict in Indian and international media. Sci. Commun..

[8] Allgaier J. (2011). The difficulty of differentiating expertise and the functions of expert sources and the necessity of studying science education on the media. Cult. Stud. Sci. Educ..

[9] Jacobson S.K., Langin C., Carlton J.C., Kaid L.L. (2011). Content analysis of newspaper coverage of the Florida panther. Conserv. Biol..

[10] McCombs M.E., Shaw D. (1972). The agenda-setting function of the mass media. Public Opin. Q..

[11] Bhatia S., Athreya V., Grenyer R., McDonalds D.W. (2013). Understanding the role of representations of human-leopard conflict in Mumbai through media-content analysis. Conserv. Biol..

[12] Killion A., Melvin T., Lindquist E., Carter N.H. (2018). Tracking half-century of media reporting on gray wolves. Conserv. Biol..

[13] Sakurai R., Jacobson S.K., Carlton J.S. (2013). Media coverage of the management of the black bear *Ursus thibetanus* in Japan. Oryx.

[14] Fernández-Gil A., Naves J., Ordiz A., Quevedo M., Revilla E., Delibes M. (2016). Conflict misleads large carnivore management and conservation: Brown bears and wolves in Spain. PLoS ONE.

[15] Nie M.A. (2002). Wolf recovery and management as value-based political conflict. Ethics Place Environ..

[16] Blanco J.C. (2017). La gestión del lobo en España. Controversias científicas en torno a su caza. Arbor.

[17] Houston M.J., Bruskotter J.T., Fan D. (2010). Attitudes towards wolves in the United States and Canada: A content analysis of the print news media, 1999–2008. Hum. Dimens. Wildl..

[18] Sáenz de Buruaga M., Canales F., Campos M.A., Noriega A., Muñoz F.J., Navamuel N. (2015). Censo Regional del Lobo (Canis lupus) en Castilla y León.

[19] Junta de Castilla y León (2017). Plan de conservación y gestión del lobo en Castilla y León: Memoria 2017.

[20] Gil L., Torre M. (2007). Atlas forestal de Castilla y León.

[21] Font Tullot I. (2000). Climatología de España y Portugal.

[22] Trouwborst A. (2014). The EU Habitats Directive and wolf conservation and management on the Iberian Peninsula: A legal perspective. Galemys, Span. J. Mammal..

[23] Junta de Castilla y León (2014). Análisis Justificativo de la Revisión del Plan de Conservación y Gestión del lobo en Castilla y León.

[24] Kuckartz U. (2014). Qualitative Text Analysis: A Guide to Methods, Practice and Using Software.

[25] Junta de Castilla y León (2016). Plan de Conservación y Gestión del Lobo en Castilla y León: Memoria 2016.

[26] Walker J.M.M., Godley B.J., Nuno A. (2019). Media framing of the Cayman turtle farm: Implication for conservation conflicts. J. Nat. Conserv..

[27] Kearny M.W., Allen M.R. (2017). Cramér’s V. The Sage Encyclopedia of Communication Research Methods.

[28] McDougall C. (1982). Interpretative Reporting.

[29] Price V., Tewksbury D., Powers E. (1997). Switching trains of thought: The impact of news frames on readers’ cognitive responses. Commun. Res..

[30] Gore M.L., Siemer W.F., Shanahan J.E., Schuefele D., Decker D.J. (2005). Effects on risk perception of media coverage of a black bear-related human fatality. Wildl. Soc. Bull..

[31] Siemer W.F., Decker D.J., Shanahan J. (2007). Media frames for black bear management stories during issue emergence in New York. Hum. Dimens. Wildl..

[32] Bisi J., Kurki S., Svensberg M., Liukkonen T. (2007). Human dimensions of wolf (*Canis lupus*) conflicts in Finland. Eur. J. Wildl. Res..

[33] Shmueli W.F., Elliot M., Kaufman S. (2006). Frame changes and the management of intractable conflicts. Confl. Resolut. Q..

[34] Montag J.M., Patterson M.E., Freimund W.A. (2005). The wolf viewing experience in the Lamar valley of Yellowstone National Park. Hum. Dimens. Wildl..

[35] Madden F. (2008). The growing conflict between humans and wildlife: Law and policy as contributing and mitigating factors. J. Int. Wildl. Law Policy.

[36] Madden F. (2004). Creating coexistence between humans and wildlife: Global perspectives on local efforts to address human-wildlife conflict. Hum. Dimens. Wildl..

[37] Abroms L.C., Maibach E.W. (2008). The effectiveness of mass communication to change public behavior. Annu. Rev. Public Health.

[38] Küng L., Picard R.G., Towse R. (2008). The Internet and the Mass Media.

